# Key determinants of health and wellbeing of dental nurses: a rapid review of over two decades of research

**DOI:** 10.1038/s41405-025-00314-y

**Published:** 2025-05-27

**Authors:** Nana Adwoa Opoku-Ansah, Jennifer E. Gallagher, Victoria Niven

**Affiliations:** Dental Public Health, Centre for Host Microbiome Interactions, Faculty of Dentistry, Oral & Craniofacial Sciences, King’s College London, London, UK

**Keywords:** Health care, Dentistry

## Abstract

**Aim:**

To review the literature on the key determinants of health and well-being amongst dental nurses.

**Method:**

A rapid review of the literature using Khangura’s approach across seven health and social science databases was conducted (2002 to 2023), through OVID and the Cochrane Library, professional and health system databases—informed by past research exploring the health and well-being of dental professionals. A two-stage review process was implemented where records were screened by the primary researcher, with a second researcher independently reviewing 10% of the papers according to agreed inclusion and exclusion criteria. Data extraction and qualitative synthesis of the included studies were conducted, and quality was assessed using a Mixed Methods Appraisal Tool.

**Results:**

Out of 4,450 identified papers, 44 underwent full-text screening and 37 studies were included for analysis. Four studies were of high quality, 13 moderate-high, 14 moderate-low and six low-quality. The overall health and well-being of dental nurses was variable and the determinants influencing well-being were grouped into micro-, meso- and macro-level factors with evidence across the three domains with the most frequently identified determinant being workplace characteristics. Dental nurses with extended duties had higher job satisfaction. There was evidence that during the COVID-19 pandemic, there were increased levels of anxiety, stress, burnout and isolation among dental nurses.

**Conclusion:**

The body of research suggests that dental nurses’ health and well-being determinants are comparable to those of other dental professionals. Dental nurses in different countries had similar determinants. It is important to recognise and improve the determinants of dental nurses’ health and well-being to support retention within the profession. Organisational and policy changes may help improve the well-being of dental nurses. Further investigation into dental nurses’ health and well-being over time is needed.

## Introduction

Dental nurses, also known as dental assistants in some countries, work alongside and support other dental team members including dentists, dental therapists and hygienists [1, 2]. In this paper, dental nurses will be used as a term to represent dental nurses and dental assistants. Dental nurses have an essential role in the delivery of care and form a significant portion of the dental team, with a ratio of dentists to dental nurses of 1:1.5 in the UK. How they are trained, their specific roles and professional registration vary from country to country, with some countries not requiring registration. In some parts of Europe, such as the United Kingdom and The Netherlands, dental nurses have a national register, whereas, in the United States and Canada, the registration and regulation of dental nurses vary according to state and province dental boards respectively [3–5].

The WHO defines health as ‘a state of complete physical, mental and social well-being and not merely the absence of disease’ [6]. Well-being in turn is defined as a ‘positive state experienced by individuals and societies which encompasses quality of life and the ability of societies to contribute to the world with a sense of meaning and purpose’ [7]. It is imperative to emphasise that employee health goes beyond the absence of illness and includes physical, mental and social well-being and practices that prioritise employee health and well-being outperform their rivals in terms of patient satisfaction, quality ratings, results, staff retention and sick leave rates suggesting a strong argument for doing so [8].

There has been well-documented evidence of high rates of stress and burnout among dentists and the importance of dentists’ health has been recognised, leading to the development of specialised programmes such as NHS Practitioner Health in England, a targeted mental health service for doctors and dentists in 2019 [9]. Before the pandemic, it appeared that healthcare workers (HCWs) were more prone to stress, burnout and depression, which can be aggravated by public health emergencies affecting their mental health and general well-being [10]. The pandemic had a profound impact on health systems in most of the world’s countries, especially in terms of the mental health and well-being of medical professionals leading the pandemic response [10] with over one-third of HCWs, reporting anxiety and sadness, with more than four times as many (from 5.2% to 21.6%) of them reported having extremely high levels of symptoms [11]. During the pandemic, safety, redeployment to support other services and uncertainty over COVID-19 guidelines were perceived as additional sources of stress and worry for the dental team [12, 13].

In both the United Kingdom and Germany, low job satisfaction is caused by a variety of issues, including emotions of helplessness, and feeling exploited, which contributed to a high turnover of dental nurses [14, 15]. It has been suggested remuneration, favourable working relationships and surroundings, training opportunities and flexible schedules should be considered to aid in both the retention and recruitment of dental nurses [14, 16]. A para-pandemic survey of dental nurses in the UK Dingle and Balmer [17] found that 65% (*n* = 655) of dental nurses reported that they have considered leaving dentistry since the COVID-19 pandemic.

In their study, Zhang et al. [18] found dental nurses’ psychological distress was influenced by their age, lower pay, longer workweeks, burnout, high levels of stress and discontent at work, a lack of leisure time, and a subpar healthcare environment [18]. Despite the call for further studies, and evidence on dental nurses’ well-being, given these risk factors, globally, there remains limited research in this field. Given the magnitude of the dental nurse workforce (global nurse workforce of ~1,290,000 [19]), their significant contribution to the dental team in various countries, the growing complexity of their jobs as well as the breadth of additional skills they possess, their needs and well-being must be investigated to avoid a mass exodus which would potentially cripple dental services [20].

Prior to COVID-19, both primary research as well as rapid reviews were undertaken on the well-being of dentists to add and to summarise evidence on the well-being of the dental workforce [21–23]. In a review of key findings relating to dentists [21, 23], the wide range of current impacts on dentists’ health and well-being were highlighted, ranging from macro-level (primarily professional regulation and healthcare system), meso-level (workplace and employment needs) and micro-level (professional relationships and personal life). In a subsequent review building on research by Salazer et al. [23], and investigating dental professionals as a whole, it was reported that factors affecting the well-being of dentists like workplace elements such as the healthcare system, occupational hazards, career level, job specification, working hours and supportive networks, were comparable to those affecting other Clinical Dental Care Professionals [23, 24].

Therefore, it was appropriate to conduct a study of the literature, determining the health and well-being of dental nurses and assessing the macro-, meso- and micro-level key determinants of well-being, mirroring the work done on dentists and other dental professionals [21–23]. However, given the challenges to health professionals, it would be critical to distinguish between pre- and para-COVID research given its impact on the well-being of the dental team.

### Aim

To review the literature on the key determinants of health and well-being amongst dental nurses.

### Objectives

1. To compare pre-pandemic and para-pandemic research findings.
2. To synthesise studies qualitatively by the determinants, in relation to macro-, meso- and micro-level influences.

## Method

A rapid review using Khangura et al.’s [25] methodological approach was conducted to search and summarise the literature. The evidence obtained was combined according to the guidelines provided by the Preferred Reporting Items for Systematic Reviews and Meta-Analyses (PRISMA) [26, 27]. This technique has been used in several papers including the reviews on the well-being of dentists and dental care professionals and provides evidence in a condensed timeframe and an understandable format, balancing the rigour employed to conduct a summary of evidence with the timetable within which the information is required [21, 23, 25, 28–30].

### Search strategy

A rigorous search of the literature between January 2002 and May 2023 was undertaken. Two decades was seen as a sufficient span to detect the evolution of well-being and its determinants. The professional and health system databases searched were Cochrane Library, Global Health, Health Management Information Consortium, Embase, PsycINFO, Journals@Ovid, Social Policy and Practise and Ovid MEDLINE(R) OVID. Search terms are presented in Supplementary Material.

### Study selection

Records identified were downloaded to Rayyan® software [31], duplicates were removed and the remaining papers were screened by the primary researcher (NA) with a second researcher (VN) independently reviewing 10% of the papers screened using the title, abstract and full text by the inclusion and exclusion criteria. Disagreements were resolved by a third researcher (JG).

### Quality assessment

A Mixed Methods Appraisal Tool was used to evaluate the quality of papers and bias risk [32]. This method of appraising studies is reliable, can assess the quality of the various types of study designs found in reviews and has been used in several similar research papers [23, 33]. This tool can be used to evaluate qualitative, mixed-methods, quantitative descriptive, nonrandomized and randomised controlled trial investigations. Each category has five core criteria, i.e. factors that are most pertinent to evaluating the methodological quality of investigations. A yes, no and can’t tell scale is used to rate each requirement combined to provide an overall quality assessment outcome.

### Data extraction

Data from the selected studies was extracted using a previously piloted data collection form comprising: the type of paper, characteristics of participants, methodology, outcome measurements, main findings and author conclusions [23, 24].

### Data synthesis

Using conventional techniques for thematic analysis in qualitative investigations, data was synthesised qualitatively by topic [34–36] with the major themes extracted. Influencing factors that were related to the pandemic were explored and compared with those not associated with the pandemic—general factors.

## Results

### Characteristics of included studies

**Fig. 1:**
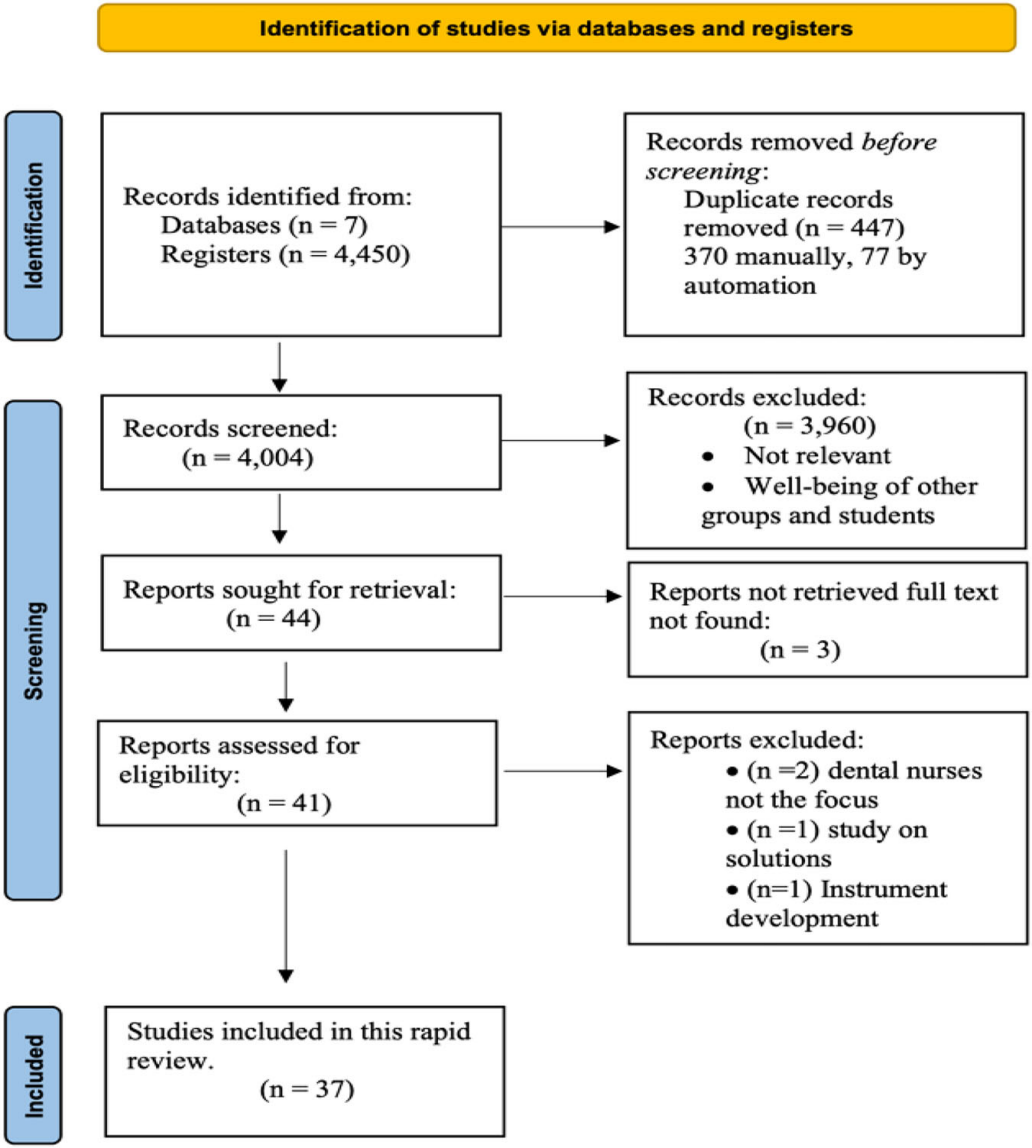
Diagram of the determinants of dental nurses’ health and well-being. PRISMA 2020 flow diagram of systematic review search process and results.

The electronic database search yielded 4450 citations with 4004 remaining after the removal of duplicates. Forty-four papers were included for full-text screening and 37 studies met the inclusion criteria and were included in this review. A detailed presentation of the process of identifying the studies is shown in the PRISMA flow chart presented in Fig. 1 [27].

There were 34 quantitative (mostly cross-sectional surveys (*n* = 31)), three qualitative, two longitudinal and one mixed-method study included in this review.

Four of the studies were identified as high quality, 13 moderate-high, 14 moderate-low and six low-quality studies using the MMAT quality tool as presented in Table 1 in the Supplementary Material.

Twenty-four (88%) of the included studies were published within the last 10 years with almost 30% of them related to the effect of the COVID-19 pandemic on dental professionals. Eighteen of the studies were conducted in Europe with eight from the United Kingdom [13, 37–43] and three from Germany [15, 44, 45]. The other European studies were from Finland [46, 47], Norway [48, 49], Sweden [50] and Slovenia [51]. There were four studies from Saudi Arabia [52–55], two each from China [18, 56], Thailand [57, 58] and Jordan [59, 60], one each from Iran [61], Turkey [62], Israel [63], the United States of America [64], Malaysia [65], Japan [66], Trinidad and Tobago [67] and Nigeria [68]. One final study provided comparative evidence of the dental workforce within the UK, New Zealand and Trinidad and Tobago [69].

Overall, the studies had clear research questions and appropriate data collection methods. All the studies used a questionnaire as part of the data collection procedure with varied methods of administration such as postal surveys [15, 38, 48, 66, 67, 69], online self-administered [37, 39, 44, 45, 55], a combination of methods [40, 43, 63], printed questionnaires [52, 57, 59, 68], via interviews, either in person, focus groups or online [41, 42, 61, 62] and computer-assisted telephone interviews [46, 47].

To augment data collected in questionnaires, studies utilised physical examinations [46, 59], photographs [64] and direct observation [65]. The findings on the current health and well-being of dental nurses (Table 1) the quality assessment scores and the key determinants of well-being of dental nurses (Table 2) of the included studies can be seen in the Supplementary Material.

### Measuring dental nurses’ health and well-being

To measure the health and well-being of dental nurses, studies examined one or more of the following areas: health (physical, emotional, psychological) (*n* = 19), job well-being (job satisfaction, hazards, burnout, work-life balance) (*n* = 15) and individual (anxiety, wellness) (*n* = 3). There were some similarities with the instruments used in the different studies with most using a combination of questionnaires. Two studies used the 22-item Impact of Event Scale-Revised and depressive symptomatology using the Patient Health Questionnaire-2 [37, 39], variations of the Generalized Anxiety Disorder Questionnaire (GAD-2, GAD-7) [40, 44], Posture Assessment Instrument [64, 65], Work Stress Inventory for dental assistants (WSI) [60, 63] and the Warr–Cook–Wall instruments [15, 43]. The Depression Anxiety Stress Scale (DASS-21) was used by four studies [45, 53, 55, 66], as was the Maslach Burnout Inventory [18, 52, 63, 70]. 78% (*n* = 29) of studies used pre-existing or validated measures, with the majority of the studies using a combination of questionnaires.

### Sample characteristics

There was evidence of research across a range of dental settings, with over 25% of the included studies conducted in university or tertiary hospitals [13, 18, 40, 52, 56, 57, 59, 62, 64, 68]. Six studies involved the use of professional/association lists [43, 45, 47, 66, 67, 69]. Six studies were conducted in a mixture of public and private settings [41, 48, 49, 53, 55, 63], four were in public facilities [42, 50, 54, 65] and primary care [37, 38, 58] and six studies had unclear settings [15, 39, 44, 46, 61, 70]. Only one study was conducted in private practice [60]. Sixteen studies focussed exclusively on dental nurses [15, 38, 53, 55, 56, 58, 60, 63, 67, 70] while the other 21 studies included other dental care professionals.

### Dental nurse’s general health and well-being

The findings of the studies on the key determinants of dental nurses’ general health, and personal and job well-being following the work of Salazar [23] are presented in Table 2 in the Supplementary Material and in three levels, namely, macro, meso and micro-level factors. Regarding the studies related to the COVID-19 pandemic, there appeared to be a worsening of the well-being of dental nurses, as evidenced by the reported increased stress and anxiety, depression and social isolation [13, 40, 45, 61, 62].

### Determinants of health and well-being among dental nurses

**Fig. 2:**
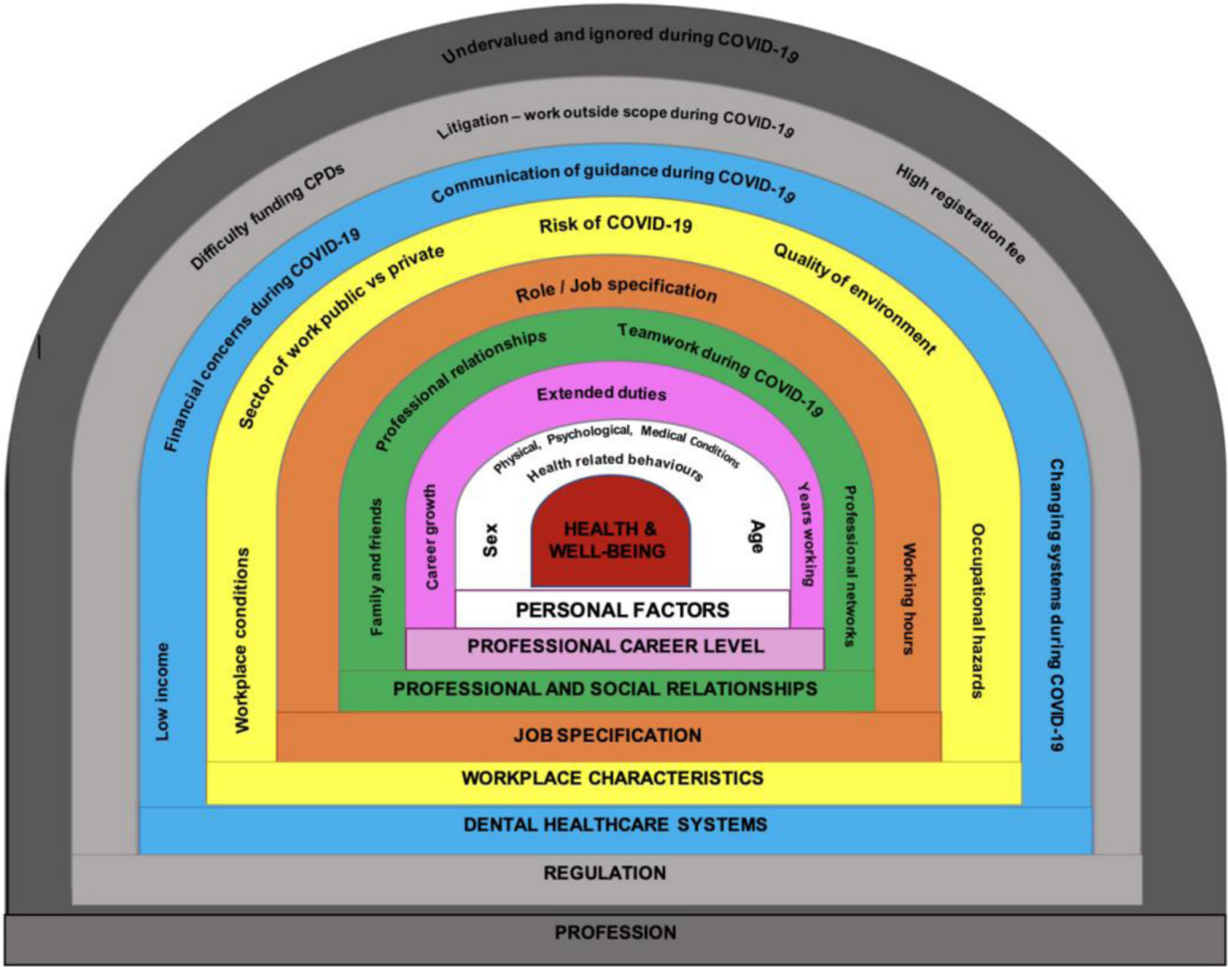
Diagram of the determinants of dental nurses’ health and well-being. Image adapted from Colonio Salazar et al., ‘Key determinants of health and well-being of dentists within the UK: a rapid review of over two decades of research’, *British Dental Journal*, 2019, Springer Nature [23]. This material is not part of the governing OA license but has been reproduced with permission.

Findings on key determinants of the health and well-being of dental nurses, namely, macro- (Dental healthcare system, regulation and profession), meso- (job specification and workplace characteristics) and micro- (personal factors, professional and social relationships, career level)-level factors, are presented in the diagram (Fig. 2) from the model by Gallagher and Colonio-Salazer [21–23].

### Micro-level determinants

#### Personal factors—(general factors)

##### Age

Increasing age was associated with upper extremity disorders, psychological distress (dental nurses > 35 years) and higher work engagement in dental nurses [18, 50, 56]. However, younger age [19–37] was associated with a higher prevalence of hand eczema among dental team members in clinics in Japan and younger workers with atopic dermatitis had a higher risk of developing hand eczema [66].

##### Sex

Although not statistically significant, it was found that more women experienced musculoskeletal pain than men (82.8% compared with 68.4%) in Slovenia whereas in Saudi Arabia, most female dental nurses were happier with their salary than their male counterparts (*p* < 0.05) [51, 53].

##### Family factors

In a Chinese study, dental nurses over 46 years faced pressures from their children going to college and elderly parents leading to lower work engagement compared with those aged 40–45 years who had children who could care for themselves (*p* = 0.001) [56].

#### Personal factors (pandemic-associated factors)

##### Age and sex

In a German study by Dreher et al. [44], dental nurses in the oldest age group (>41 years) health and well-being were significantly associated with an increased perceived risk of COVID-19 infection (OR 1.64 (95% CI 1.13–2.39)) whereas, in Norway, women reported being significantly more concerned about contracting the COVID-19 virus than their male counterparts [49].

##### Health-related behaviours

Dental nurses in Germany and the UK who had a higher self-rating of their health were significantly more likely to feel sufficiently prepared for dealing with patients with COVID-19 however, there were high levels of anxiety around personal protection and health (1.54 (95% CI 1.12–2.12)) [41, 44]. Dental nurses in China who regularly exercised were found to have statistically significant higher work engagement [56].

##### Family factors

In an Iranian study, staff with relatives who died from COVID-19 were 2.47 times more likely to have anxiety than those who did not [61].

#### Professional career level (general factors)

In China, the more advanced in their careers dental nurses were, the higher work engagement they had due to more experience and stable family life; however, in Saudi Arabia, when compared to other dental professionals, dental assistants (dental nurses) had the lowest level of personal accomplishments in terms of their self-assessment of their proficiency and productivity (*p* = 0.048) [52, 56].

##### Years working as a dental nurse

Dental assistants (dental nurses) in Norway with more work experience had a statistically significant increased prevalence of symptoms consistent with cognitive malfunction like memory and psychosomatic symptoms [48].

##### Extended duties/additional skills

Within the UK, most dental nurses (87%) agreed that continuous professional development courses had improved their skills and expanded their scope of practice (e.g. taking impressions, radiographs and applying fluoride varnish) [43]. In Scotland, extended-duty dental nurses were completely (28.2%) or very (39.7%) satisfied with their role and listed ‘resources’ and ‘supportive colleagues’ as facilitators to their success [38].

#### Professional and social relationships (general factors)

Dental nurses felt overworked and were unsatisfied with their relationships in Thailand, with a Saudi study echoing this and reporting that only 31.9% of dental nurses were satisfied with their professional and personal lives [53, 58].

#### Professional and social relationships (pandemic-associated factors)

At the height of the COVID-19 pandemic in the UK, self-isolation and social distancing put further strain on these relationships [13, 40]. Nevertheless, in one qualitative study of staff working in Urgent Dental Care Centres (UDCs) in England, dental nurses felt that their professional relationships were strengthened and they learned from and appreciated each other [41].

### Meso-level determinants

#### Job specification (general factors)

Dental nurses in Thailand with numerous and non-specific roles reported more internal conflict which increased their stress and reduced their effectiveness [58].

##### Working hours

Dental nurses who worked fewer hours (<20 h/week) reported being more satisfied with their work (and income) compared to those working longer hours [15]. There was a similar finding in Saudi where male dental nurses working >8 h per day were less satisfied than those working less than 8 h (*p* = 0.01) [53].

#### Job specification (pandemic-associated factors)

During the pandemic, only 49.6% of dental assistants felt well-informed about interacting with patients with the virus with 87.6% unsure of the proper course of action to take and those that were redeployed and working in UDCs were concerned about working outside of their field of expertise, being assigned to a setting with higher risk (wards with patients positive for COVID), worries about the scarcity of Personal Protective Equipment (PPE) and feeling vulnerable to litigation [40, 41, 44].

##### Working hours

Working two back-to-back shifts daily was significantly associated with moderate levels of Post Traumatic Stress Disorder symptoms [61].

#### Workplace characteristics (general factors)

##### Private/Public sector

Dental nurses working in the private sector in Saudi Arabia were more satisfied than those employed in the public sector [53].

##### Work environment

Dental nurses reported being somewhat satisfied with their jobs in Jordan (53.5%) [60] which contrasted with the findings by Naidu [67] in Trinidad, where unsatisfactory working conditions for dental nurses were reported. Additionally, studies in Israel and China reported that there was psychological distress in dental nurses which was linked to lower pay, longer workweeks, burnout, high job stress, lower job satisfaction, regret over career choice and violent events in the hospital [18, 63].

##### Occupational hazards

In a Finnish study, 25% of dental nurses reported work-related dermatoses on their hands, forearms or face [46] while in Jordan, compared to controls (third-year dental students- preclinical), they had poorer hearing in the left ear which was statistically significant at the higher frequencies but not at lower frequencies [59]. Additional occupational hazards include musculoskeletal pain (46.8% of Thai dental nurses), daily use of methacrylates which was related to a significantly increased risk of adult-onset asthma (adjusted OR 2.65, 95%; CI 1.14–7.24), nasal symptoms (dose-response) (1.37, 1.02–1.84) and work-related cough or phlegm (1.69, 1.08–2.71) [44, 47, 57].

#### Workplace traits (factors related to the pandemic)

Concerning work during the pandemic, there was significant anxiety among dental staff in Norway, Turkey and the United Kingdom about restarting aerosol-producing procedures. Additionally, the constantly changing guidelines and increased patient demands in the UK and a higher risk of contracting COVID-19 in Germany were factors that negatively impacted the well-being of dental nurses [13, 41, 44, 49, 62].

### Macro-level determinants

#### Dental healthcare systems (general factors)

##### Regulation

While dental nurses in Trinidad and Tobago were confused about the legislation regarding private practice, in the UK, dental nurses were unhappy about the cost of registration, with 74% finding the charge too expensive even though they agreed with completing Continuing Professional Development (CPD) and compulsory registration [43, 67].

#### Dental healthcare systems (pandemic-associated factors)

##### Profession

There was dissatisfaction with communication from the health system in England at the start of the pandemic [41]. Additionally, dental nurses in the UK report difficulty in paying for CPD with some opting to attend only free courses [43]. Dental nurses in Germany and Saudi Arabia were dissatisfied with their income and were uncertain about their financial situation during the pandemic [44, 53].

Comparing the pre-pandemic and para-pandemic well-being of dental nurses, the pandemic increased stress, anxiety and redeployment, causing a decline in the health, especially mental health, and well-being of dental nurses [13, 40, 41, 61].

## Discussion

This review is the first rapid review of the health and well-being of dental nurses worldwide. It combined available studies over the last two decades with information on the health and well-being of dental nurses in 19 countries. With the examination of the health and well-being of dental nurses, as well as the determinants across the micro-, meso- and macro-level factors outlined by Gallagher et al. [21], Salazar et al. [23] and Kaki et al. [24], finding that most papers reported on meso-level factors both in pre- and para-pandemic papers, with workplace characteristics being the most important factor followed by job specification. Micro-level factors such as  professional and social relationships, as well as personal factors also influenced the reported health and well-being of dental nurses.

There was limited information that was specific to dental nurses/assistants, especially on the determinants of their health and well-being. Most of the studies looked at the wider dental team, of which dental nurses are a part, with a minority (16 (43%))of the included studies did not include any other dental group [15, 38, 53, 55, 56, 58, 60, 63, 67].

Regarding the well-being of dental nurses worldwide, those from Trinidad and Tobago and Nigeria reported lower satisfaction compared to those in the United Kingdom and New Zealand. This could have been due to the challenging working conditions such as workplace violence and low salaries [67, 68]. In more developed countries, there was evidence of  higher satisfaction but low income, occupational hazards (musculoskeletal pain, skin conditions) and burnout [15, 50, 51, 54, 66] were factors negatively impacting well-being.

The methods used in this study were adopted from previous similar research and followed the methodology of systematic reviews, ensuring reproducibility [23, 25]. The screening was undertaken by two blinded researchers with 100% agreement increasing confidence in the included studies. However, the results of this review should be interpreted cautiously given the quality of the studies and the lack of longitudinal research in this field. It should also be noted that only studies in the English language were included and some of the studies did not use validated instruments.

Overall, in the pre-pandemic literature, dental nurses reported that they were satisfied with their health and well-being with studies reporting similar determinants. Well-being was evidenced by high levels of job satisfaction, with one study in Malaysia [65] reporting 93.4% satisfaction, a lack of distressing psychological symptoms or low rates of distress (20.8%) and high levels of job effectiveness [38, 45, 58], notably when they had gained extended skills [38, 43].

In both the pre- and para-pandemic literature, the most common micro-level factor was personal factors followed by professional and social relationships and lastly professional career level. Amongst  personal factors, age, sex and family were most prevalent while isolation from family and friends and improved teamwork during COVID-19 were the most noted among the professional and social relationship determinants [38, 40, 44, 53].

Regarding meso-level factors, dental nurses in European, Asian and Middle Eastern countries, reported higher job satisfaction. Occupational hazards included musculoskeletal disorders, dermatoses and noise-related hearing issues. These as well as the increased uncertainty, evidence of high levels of stress and anxiety and change in workplace procedures during the COVID-19 pandemic influenced the health and well-being of dental nurses [41, 46, 57, 59].

Macro-level factors were infrequently reported and were associated with the professionalism of dental nursing including the high cost of registration, the challenge of funding CPD [43, 67] and the lack of suitable continuing education courses in Trinidad and Tobago [43, 67], together with poor communication in the early phase of COVID-19 in England [41].

Workplace characteristics were the most prevalent determinant echoing the findings in reviews on other dental professionals [23, 24]. Low income and burnout were negative influences while societal support, feeling appreciated and enhanced teamwork were positive influences impacting the well-being of dental nurses. Dental nurses with extended duties reported higher satisfaction which could be attributed to higher income and feeling more fulfilled in their career [15, 38, 41, 56, 65].

Comparing pre- and para-COVID papers, there were notable factors that affected the health and well-being of dental nurses, with the increased stress and anxiety causing a decline in their health, especially mental health, and well-being of dental nurses [13, 61]. Similar to other health professionals [10, 11], the pandemic period seemed to stimulate research into dental nurses’ health and well-being as part of the dental team as evidenced by the more recent papers.

The determinants of the well-being of dental nurses found in this review are similar to those reported in reviews on dentists and other members of the dental team [23, 24]. These other reviews, however, were specific to the UK while this one on dental nurses is worldwide, adding to the knowledge of dental nurses. These comparable results suggest that worldwide, although there may be differing dental health systems, the determinants of the health and well-being of dental nurses are similar. These findings suggest that opportunities for career development should be encouraged given that dental nurses with extended duties report a greater sense of well-being [38, 43]. Additionally, resources available to dental nurses to improve their stress and mental health nationally and in their workplaces should be improved and made more accessible and acceptable.

## Conclusion

Generally, dental nurses reported positive job satisfaction, although there was a slight variation from country to country with working hours, environment and occupational hazards being the main themes. During the pandemic job specification (working in high-risk environments) and availability of PPE were the main concerns of dental nurses [40, 41, 44, 53, 67] The majority of the studies focussed on meso-level determinants namely workplace characteristics. Dental nurses with higher professional levels attained through extended duties had better job satisfaction than those without. The results echoed the findings from reviews on other members of the dental team. Furthermore, there were notable stresses reported due to the pandemic which appears to have affected the health and well-being of dental nurses.

Dental nurses form a large proportion of dental professionals, perform essential duties within the dental team and are vital to providing quality dental care. Given their significance, it is important to research their health and well-being, especially due to the negative impacts of COVID-19, and the lack of available research worldwide is concerning. To sustain and improve dental services, it is essential to maintain the health and well-being of this population. It is prudent that employers, policymakers and regulatory authorities acknowledge the determinants and foster a work environment that supports the health and well-being of dental nurses and reduces stress. More longitudinal studies focusing on dental nurses incorporating qualitative research should be conducted. This study did not require ethical approval as it was a rapid review of existing published literature.

## Supplementary information

Supplementary Tables

## Data Availability

The data supporting the findings in this paper are available from the corresponding author upon request.
