# Supplementary material for: Key determinants of health and wellbeing of dental nurses: a rapid review of over two decades of research
Source: BDJ Open. 2025 May 27;11:53. doi: 10.1038/s41405-025-00314-y (PMC12117156; doi:10.1038/s41405-025-00314-y)
Supplement: Supplementary file 1 — Supplementary Tables [file 41405_2025_314_MOESM1_ESM.pdf]

Supplementary table
Table 1 Summary of the Current Status of Dental Nurses' Health and Well-being of the Included Papers

Colour code key - **Pre Covid-papers - blue heading**
Para Covid papers – Grey heading
Para pandemic papers that did not focus on the pandemic- Red borders
Papers where dental nurses cannot be separated – Yellow highlight

Study Author/ Date	Country/ Year of study	Professional Group	Setting	Study type Health/ Wellbeing instrument	Findings on current health and well being	Overall judgement/ MMAT score
NAIDU, R. S. et al / 2002	Trinidad and Tobago (T&T)<br><br>Did not specify	Dental nurse (n=38)	Enrolled by the Trinidad and Tobago dental council	Cross sectional<br><br>Questionnaire not specified	Low level of job satisfaction with a mean value of job satisfaction of 5.2 (SD =2.3). The least rewarding were 'poor salary and work conditions' and 'lack of a career path'	Nurses showed desire in further education and were confident in performing current duties, but overall work satisfaction was poor.<br><br>5 – Moderate High
ALANKO, K et al / 2004	Finland<br><br>Did not specify	Dental nurses n=799 were interviewed and n=328 were examined	Nurses in Helsinki Metropolitan area	Cross sectional<br><br>Tuohilampi questionnaire for contact dermatitis and atopy/ Exam	41% of dental nurses reported work-related dermatitis on their hands, forearms, or face. The frequency of hand and facial dermatoses was significantly associated with atopy. Allergies were from rubber compounds, natural rubber latex in gloves, and plastic materials used in restorations (methacrylates).	Out of 107 nurses, there were 29 cases of allergic contact dermatitis, 15 of contact urticaria, 12 of irritant contact dermatitis, and 1 case of onychomycosis.<br><br>4 – Moderate High
LINDFORS, P. et al / 2006	Sweden<br><br>Did not specify	Dentists, dental hygienists, and dental nurses 57% (n=532)	Employees of the Swedish Dental Public Service	Cross sectional<br><br>Developed a questionnaire on work characteristics, fatigue, and health problems	79% of dental nurses had upper extremity disorders (UED). Those with UED had significantly higher scores on physical work environment and work-related worries, but lower scores on support than those without. Dentists had higher scores on physical load than nurses (p=0.0001). Nurses had lower scores than others on influence at work (p<0.01)	Female dental health professionals remain at high risk of getting UED despite advancements in the physical work environment and ergonomics in dentistry.<br><br>2 – Low

Study Author/ Date	Country/ Year of study	Professional Group	Setting	Study type Health/ Wellbeing instrument	Findings on current health and well being	Overall judgement/ MMAT score
NAIDU, R. et al /2006	Trinidad and Tobago, the United Kingdom, and New Zealand.<br><br>Did not specify	Dental therapists in UK and New Zealand, Dental nurses in Trinidad 4.4% (n=50)	Nurses in Trinidad and Tobago and dental therapists in the UK and New Zealand	Cross sectional<br><br>Questionnaire not specified	Trinidad nurses had significantly lower career satisfaction than the other two groups. 59% of the therapists in NZ, 52% in the UK and 39% in Trinidad felt unvalued. Therapists in NZ were almost twice as likely to express high levels of career satisfaction in comparison to the other two countries.	Nurses in Trinidad and Tobago had the least level of job satisfaction. Feeling valued in the dental team was correlated with career satisfaction in all three nations.<br><br>2 – Low
JAAKKOLA, M. et al / 2007	Finland<br><br>Did not specify	Dental assistants (n=923)	Registered dental assistants in Helsinki	Cross sectional<br><br>Questionnaire used in previous study (Leino et al., 1997)	Daily exposure to methacrylate (64% of dental assistants) had a significantly increased risk of adult-onset asthma adjusted OR 2.65 (95% CI 1.14-7.24) The risk of nasal symptoms was also clearly increased among atopic dental assistants (2.11, 1.08–4.19)	The findings imply that methacrylate exposure is a significant work-related risk for dental assistants.<br><br>4 – Moderate High
HILT, B. et al / 2009	Norway<br><br>Did not specify	Dental assistants (n=608)	Public and private dental assistants in three counties	Cross sectional<br><br>Norwegian version of 'Euroquest " questionnaire and records of urine mercury levels	Dental assistants may suffer from long-term cognitive effects, seen as memory problems, neurological symptoms, and fatigue when compared to controls. Significant relationship between the sum of the symptom scores and the maximum urine value and number of years worked respectively, disappeared with age in the model	There may be long-term cognitive repercussions for certain dental assistants who were exposed to mercury from dental amalgam in the past. Causality cannot be inferred due to the methodology used<br><br>2 - Low
KHADER, Y. S. et al/ 2009	Jordan<br><br>10 months	Dental assistants (n=542)	Private dental clinics in Jordan	Cross sectional<br><br>Work Stress Inventory for Dental Assistants -WSI-DA	About 61% of dental assistants reported that they had stress however 53.5% were fairly well-satisfied with their job.	About half (44.7 percent) of participants reported that they did not find "real enjoyment in their job"<br><br>4 – Moderate High

Study Author/ Date	Country/ Year of study	Professional Group	Setting	Study type Health/ Wellbeing instrument	Findings on current health and well being	Overall judgement/ MMAT score
WIJARANAPHITI, S. et al / 2009	Thailand<br><br>Did not specify	Dental nurses (n=326)	Primary care units (310) of nineteen provinces	Cross sectional<br><br>Developed and validated a questionnaire on roles	Performance effectiveness of dental nurses was high. Effectiveness depended on job characteristics, motivation, Affiliation, Acceptance and Self-actualization, and role conflict between internal standard and role conflict between several roles.	There was a positive relationship between motivation and performance effectiveness at a low level.<br><br>3 – Moderate Low
DAJPRATHAM, P. et al /2010	Thailand<br><br>2008-2009	Clinical instructors, post grad students, dental assistants 46.8% (n=74)	Faculty of Dentistry Mahidol University	Cross sectional<br><br>Questionnaire not specified and body diagram	46.8% of dental assistants reported musculoskeletal (MS) pain at least one site. Dental assistants reported pain in the knee, leg and foot compared to other groups.	The most common type of MS pain was cervico-brachial discomfort, and their work status was linked to their MS pain issues.<br><br>3 – Moderate Low
AZODO, C et al / 2011	Nigeria<br><br>2009	Dentists, dental nurses 18.1 (n=32), technologists, therapists, record officers	Oral Health centres of four university teaching hospitals	Cross sectional<br><br>Questionnaire not specified	The prevalence of violence in Nigerian Oral healthcare centres was 31.9%. There was no statistically significant difference in the prevalence of violence against dentists and dental auxiliaries. Violence was most associated with long wait times (27.3%)	The well-being of dental practitioners was significantly impacted by the high incidence of workplace violence against them at oral healthcare facilities.<br><br>2 - Low
SAMAT, R. A. et al / 2011	Malaysia<br><br>2010	Dentists, dental nurses 30% (n=105), dental technicians, dental surgery assistants 37.7% (n=132)	Government dental workers in North- Eastern State of Malaysia.	Cross sectional<br><br>Branson Posture Assessment Instrument (BPAI) and direct observations of respondents	93.4% of respondents were satisfied with their job and 97.4% received support from their employers and peers (98.6%). 48% of dental assistants and 44.8% of dental staff nurses reported back pain. In the adjusted model, significant factors were poor posture (OR 3.52 95% CI 2.22, 5.59) and being an auxiliary (OR 3.63 95% CI 1.81-3.60).	The prevalence of back pain was high. Its two main risk factors were having bad posture and being a dental auxiliary.<br><br>3 – Moderate Low

Study Author/ Date	Country/ Year of study	Professional Group	Setting	Study type Health/ Wellbeing instrument	Findings on current health and well being	Overall judgement/ MMAT score
MINAMOTO, K. et al /2012	Japan<br><br>2011-2012	Dentists, hygienists, technicians, assistants 14% (n=75) and receptionists	Dental clinics organised in a city dental association	Cross sectional<br><br>Nordic Occupational Skin Questionnaire 2002 and physical examination	Most important risk factors for the 1-year prevalence were history of atopic dermatitis [(OR) 4.7, 95% confidence interval (CI): 2.2–8.8], asthma and/or allergic rhinitis (OR 2.0, 95%CI: 1.3–3.0), dry skin (OR 1.7, 95%CI: 1.1–2.7), shorter duration of work (OR 2.0, 95%CI: 1.2–3.5 for up to 10years versus >20years), and washing hands >10 times per day (OR 1.6, 95%CI: 1.0–2.5).	Dental professionals in Japan experienced a high incidence of hand eczema due to their heavy exposure to allergens like rubber and methacrylates, as well as wet work. It seems that little was known about skin protection techniques.<br><br>3 – Moderate Low
TURNER, S. et al / 2012	United Kingdom<br><br>Did not specify	Dental nurses (n=267)	UK private and NHS practices, community services, dental hospitals (GDC)	Cross sectional<br><br>Warr-Cook-Wall scale	44% of dental nurses were satisfied with their job but about 25% intended to leave the profession. Reasons were GDC registration fee, low pay and poor working conditions	Widespread complaints about registration and CPD cost and possible high rate of attrition from the profession due to low salaries.<br><br>3 – Moderate Low
Yousra H.AL JAZAIRY et al / 2014	Saudi Arabia<br><br>2013	Dental assistants (n= 498)	Public and private institutions	Cross sectional<br><br>Dental assistant Satisfaction Survey	Dental assistants were generally considerably satisfied with their jobs (76.5%). non-Saudi dental assistants were more satisfied with their professional and personal life than the Saudi dental assistants (P<0.05) .	Professional and personal life, quality of service, perception of income, prestige and self-respect are important factors for job satisfaction.<br><br>5 – High
GNICH, W. et al / 2014	United Kingdom (Scotland)<br><br>2011	Extended duty dental nurses (n=174)	General dental practices currently delivering 'childsmile'	Cross sectional<br><br>Questionnaire about role satisfaction was developed and piloted	High levels of role satisfaction among Extended Duty Dental Nurses. Satisfaction as an EDDN was associated with the frequency of FVA (r=0.278, p<0.001)those who found the training helpful were more likely to apply varnish (r= 0.300, p<0.001)	High levels of role satisfaction While some saw a lack of compensation, nurses welcomed their new abilities and using them increased job satisfaction.<br><br>4 – Moderate High

Study Author/ Date	Country/ Year of study	Professional Group	Setting	Study type Health/ Wellbeing instrument	Findings on current health and well being	Overall judgement/ MMAT score
HAKANEN, J. et al / 2014	Finland<br><br>Did not specify	Dentist-dental nurse dyads<br>(n=470)	Dentist-dental nurse dyads in Finland	Cross sectional<br><br>Finnish version of the Maslach Burnout Inventory	Exhaustion transferred from dentists to dental nurses only when collaboration was frequent and dental nurses perceived the collaboration as friendly (b = 0.29, p<0.001) or consisting of mutual feedback.	Nurses are protected from tiredness by friendliness and mutual feedback.when dentists are worn out and cooperation is common, this may become risk factors for exhaustion.<br>4 – Moderate High
GOETZ, K et al / 2016	Germany<br><br>2006-2011	Dental nurses<br><br>(n=612)	Practices using European Practise Assessment	Cross sectional<br><br>10 item Warr-Cook-Wall job satisfaction scale	Nurses had a high level of job satisfaction and were highly satisfied with 'colleagues' and 'job variety' but unsatisfied with 'income' and 'work recognition'	Work atmosphere of nurses had the most impact on working condition and job satisfaction.<br><br>3 – Moderate Low
AL-OMOUSH, S et al / 2019	Jordan<br>Did not specify	Dentists, technicians, assistants n=23	University hospital	Cross sectional<br><br><br>Physical examination	Hearing impairment was higher among dental assistants than the control group. They had statistically poorer hearing in the left ear at the higher frequencies (4000, 8000 Hz) but not at the lower frequencies.	In the dental team, dental assistants and technicians were impacted by noise pollution the most.<br><br>4 – Moderate High
HAMASHA, A. et al / 2019	Saudi Arabia<br><br>2018	Dentist, dental assistant 41.4% (n=82) dental hygienist, dental technicians	Dental staff in National Guard Health Affairs (NGHA)	Cross sectional<br><br>Korean Dentist Satisfaction Survey(translated with pilot study)	The mean score of satisfaction was low (2.9/5). Highest mean score was for quality of service then prestige and self-perception, professional and personal life and income and job security. Saudis were more satisfied than non-Saudi in professional and personal life (p=0.01)	In general, the dental workforce had low levels of job satisfaction.<br><br><br>3 – Moderate Low
SCEPANOVIC, D. et al / 2019	Slovenia<br><br>Did not specify	Dentists, dental technicians, and dental assistants 3.4% (n=3)	Densafe Congress	Cross sectional<br><br>Cornell MS Discomfort Questionnaire and body maps	MS pain among dental practitioners was in the neck, upper back, shoulder, and right lower back. MS pain is most common in general dentists, followed by dental specialists, dental assistants, and dental technicians. Participants who regularly performed low intensity exercise had the least pain	Results showed high prevalence of Musculoskeletal pain among Slovenian dental professionals.<br><br><br>2 - Low

Study Author/ Date	Country/ Year of study	Professional Group	Setting	Study, Health/ Wellbeing instrument	Findings on current health and well being	Overall judgement/ MMAT score
UZIEL, N. et al / 2019	Israel<br><br>2014-2015	Dental assistants (n=299)	Dental assistants in Israel (nationwide)	Cross sectional<br><br>Maslach Burnout Inventory – MBI-HSS Human Services (MBI), Work stress inventory for dental assistants	18% of the assistants had a high to very high level of burnout. The most stressful work stress factors were income, workload, and work hazards. Regarding burnout, there was a moderate level of emotional exhaustion (EE) (21.86 ± 12.55) and a low level of depersonalization (4.51 ± 5.85), but a high level of personal accomplishment.	Dental assistant burnout and professional stress are significant issues that may have an impact on the health of dental staff members as well as their patients.<br><br>3 – Moderate Low
ALZAHEM, A et al / 2020	Saudi Arabia<br><br>Did not specify	Consultants, residents, and dental assistants 25.42% (n=45) Lab tech and hygienists	Dental department of tertiary medical complex	Cross sectional<br><br>Psychological Stress Measure-9 and Maslach Burnout Inventory-Human Services Survey	Dental Assistants were not as stressed as other dental groups but had a low level of personal achievements. Consultants were the most emotionally exhausted group followed by assistants.	Speciality, sex, age, marital status was not risk factors for stress and burnout. Patient care may be impacted by extreme stress, emotional exhaustion and unfulfilled accomplishment.<br>4 – Moderate High
MAHENDRAN, K. et al / 2020	United Kingdom<br><br>2020	administrative dental nurses 50% (n=60) , dental technicians and dentists	Dental department in Guy's Hospital	Mixed methods<br><br>Interviews and Generalised Anxiety Disorder Assessment (GAD-7)	16.7% of respondents displayed severe symptoms of generalised anxiety, while 53.3% displayed some symptoms. Over half the respondents reported high anxiety over redeployment to other departments. Mean GAD-7 score was 8.15 with dental nurses most affected.	Dental workers experienced severe psychosocial effects and high levels of anxiety during the COVID 19 pandemic.<br><br>4 – Moderate High
PARTIDO, B. et al / 2020	United States<br><br>2019	Dentists (n=21) and dental assistants (n=17)	The Ohio State University College of Dentistry Dental Faculty Practice	Cross sectional Modified-Dental Operator Posture & Rapid Upper Limb Assessment	Mean trunk scores for dental assistants were significantly higher than for dentists. The total M-DOPAI score for dentists was correlated to the head while for assistants it was the trunk.	Dental assistants had hunched trunk postures and dentists had compromised head postures.<br><br>3 – Moderate Low
RADFORD, P. & MCKAY, A. / 2020	United Kingdom<br><br>2019-2020	Dental nurses (n=12)	CDS clinics in Barnsley,Doncaster, Rotherham, treatment with Nitrous Oxide	Qualitative Semi structures interview	Nurses were highly motivated by patient experience and perceived that they have a vital role in the safety and behavioural management of patients and their parents. Pressures were from referring dentists and parents of patients.	RA-trained dental nurses felt that they had a crucial role in patient and parent behavioural management.<br><br>4 – Moderate High

Study Author/ Date	Country/ Year of study	Professional Group	Setting	Study,Health/ Wellbeing instrument	Findings on current health and well being	Overall judgement/ MMAT score
DREHER, A. et al/ 2021	Germany<br><br>2020	Dental assistants (n=1481)	Dental, maxillofacial and orthopaedic practices	Cross sectional<br><br>Developed a pandemic related, patient health and Generalised Anxiety disorder questionnaires	Dental Assistants were ill prepared, stressed about the pandemics temporal scope and financial implications. DAs with good self-rated health felt more prepared for patients with SARS-CoV-2 (1.54 (95% CI 1.12 to 2.12)), while DAs who reported suspected cases among their colleagues felt less prepared (0.63 (95% CI 0.44 to 0.91))	DA's were ill prepared for the pandemic. Those with symptoms of anxiety or depression felt particularly burdened<br><br>5 - High
FREEMAN, R. et al/ 2021	United Kingdom (Scotland)<br><br>2020	Dental trainees, primary dental care staff<br><br>(n=53)	Dental care staff with NHS Education for Scotland account	Cross sectional<br><br>Professional Preparedness for Practice Scale, Impact of Event Scale-Revised, Patient Health Questionnaire-2.	Staff with depressive symptoms were less prone to developing emotional exhaustion but those less prepared and with COVID-19 trauma more prone. The average baseline fatigue score was 5.36, 95% CI: 4.27-6.26), average slope 0.13, 95% CI: 0.07 to 0.19, $p<0.001$). Over 10 weeks, the respondent increased their fatigue score by 1.3 units.	Consistency with a job demands-resources model of burnout development.<br><br>4 - Low
HUMPHRIS, G. et al / 2021	United Kingdom (Scotland)<br><br>2020	vocational dentists hygienists trainee dental nurses (118, 29%) dental nurses 82(38%).	Dental trainees and primary dental care staff in Scotland	Cross sectional Professional Preparedness for Practice, Maslach Burnout, Impact of Event Scale Patient Health Questionnaire-2	36% of primary care staff were not prepared in taking care of their health ($p<0.05$) and stress levels, over half of them were not prepared financially for the effects of the pandemic and they experienced greater emotional exhaustion than trainees. Depressive symptomology was 27%	Potential advantage of providing staff support and actions to help dental professionals prepare for the COVID-19 pandemic's high uncertainty phases.<br><br>3 – Moderate Low
KIRLI, M. & KIRLI U. /2021	Turkey<br><br>Did not specify	Dentists, dental nurses 21% (n=17) Data entry and cleaning staff	University dental clinic	State-Trait Anxiety Inventory (STAI)	Restarting high risk procedures for COVID-19 infection increased anxiety levels of dental employees significantly (mean state anxiety scores 42.6 vs. 49.0; t: 3.8; $P < 0.01$). Increases in state anxiety was significant in females, nurses, dentists working in departments with more aerosol-generating procedures.	Restarting high risk procedures during the pandemic increased stress in staff that already had high levels of anxiety<br><br>3 – Moderate Low

PARA COVID						
**Study Author/ Date**	**Country/ Year of study**	**Professional Group**	**Setting**	**Study type Health/Wellbeing instrument**	**Findings on current health and well being**	**Overall judgement/ MMAT score**
MEKHEMAR, M. et al/ 2021	Germany<br><br>2020-2021	Dental nurses (n=252)	Nationwide cross-sectional survey	Cross sectional<br><br>Depression Anxiety Stress Scale (DASS-21) and Impact of Events Scale Revised Instruments (IES-R) surveys	Nurses presented no distressing or mild mental effects of COVID-19 as estimated by the Impact of Events Scale-Revised (IES-R) and the Depression Anxiety Stress Scales (DASS-21) instruments. Significant effects of financial aspects, systemic immunodeficiency conditions, and having children on mental stress, depression and anxiety of nurses.	All psychological variables showed generally mild or normal findings.<br><br>3 – Moderate Low
PLESSAS, A. et al / 2021	United Kingdom<br><br>2020	Dentists and dental nurses 23% (n=9)	Urgent Dental Care centres in community, hospital and general dental practice across England	Qualitative<br><br>Phone/internet interview	Working within UDCs often generated multifactorial stress and anxiety, particularly when there were concerns over safety and operational challenges. Poor communication and inconsistent advice were negatives while greater team unity and a strong sense of professional responsibility were positives. Coping strategies during covid include mindfulness activities, morning huddles.	Staff had physical and emotional difficulties that were made worse by an environment devoid of support, frequently as a result of poor leadership.<br><br>5 - High
SANDHU, B. K. et al / 2021	United Kingdom<br><br>2020	Dental nurses (60%, n = 24), speciality doctors speciality registrars, dental core trainees and one consultant	Royal National ENT and Eastman Dental Hospitals	Qualitative<br><br>Focus group discussion	key themes among dental nurses were anxiety, safety concerns, teamwork, family and redeployment. The themes of anxiety and safety were further explored, identifying participant discussion of feeling isolated, confusion, and specific concerns about PPE and transport to work.	The themes extracted from this research provide justification for creating support systems for the dentistry workforce in the United Kingdom.<br><br>4 – Moderate High
UHLEN, M. et al / 2021	Norway<br><br>2020	dental specialists, general dental practitioners, dental hygienists and dental assistants (n=1237)	Public and private clinics nationwide	New questionnaire on health service management, risk perception and psychological impact of COVID	71.9% of dental workers were concerned of becoming infected, 85.4% infecting others and/or of their family becoming infected (76.9%). Those who treated patients felt significantly more insecure about whether they were infected or not.	The pandemic had a significant psychological effect on dental staff whether or not they were involved in clinical care.<br><br>5 - High

Study Author/ Date	Country/ Year of study	Professional Group	Setting	Study type Health/ Wellbeing instrument	Findings on current health and well being	Overall judgement/ MMAT score
WANG, Y. et al / 2021	China<br><br>2020	Dental nurses (n=215)	School and Hospital of Stomatology, China Medical University	Cross sectional<br><br>Utrecht Work Engagement Scale, Nursing Stress Scale Work-related Acceptance Action, Scale of Perceived Social Support	Work engagement was positively associated with perceived social support, psychological flexibility and subjective well-being but negatively correlated with job stress. The higher job stress is, the lower work engagement is.	Dental nurses had an acceptable level of work engagement in terms of vigour, dedication, and absorption.<br><br>4 – Moderate High
ZHANG, Y et al / 2022	China<br><br>2021	Dentists and dental nurses 38.6% (n=1165)	180 stomatology hospitals, dental departments in general hospitals, and dental clinics in 11 Chinese provinces	Cross sectional<br><br>Chinese General Health, Maslach Burnout inventory, Consultant Mental Health Questionnaires	20.8% of dental nurses had psychological distress and they had more risk factors than dentists. The distress was associated with increasing age, low income, working hours, commute time, burnout stress, lower job satisfaction and career regret, similar to dentist ($p<0.05$) except age.	Among dental medical professionals, psychological stress was somewhat common; dentists were more likely to experience it than dental nurses. While nurses had more risk factors.<br><br>4 – Moderate High
BANAKAR, M. et al / 2023	Iran<br><br>2022	Dentists, hygienists, assistants 18.7% (n=119), dental students	Care providers from different parts of Iran	Cross sectional<br><br>Insomnia Severity Index, Hospital Anxiety and Depression Scale, Global Psychotrauma screening questionnaires	Prevalence of insomnia, anxiety, depression, and moderate to high levels of PTSD among dental providers was 31.3%, 40.8%, 54.9%, and 43.4%, respectively. DAs (OR=3.20, 95% CI: 1.15–8.94) had higher odds of being included in the high level of PTSD symptoms. COVID-19 infection and death in relatives were 2.89(95% CI: 1.39–6.02) and 5.59 (95% CI: 2.69–11.49) times more likely to show high levels of PTSD.	Amid the COVID-19 pandemic, there was a comparatively high frequency of mental health disorders among dental professionals in several locations in Iran.<br><br>4 – Moderate High

KOTHA, S. B. et al/ 2023	Saudi Arabia<br><br>2022	Dental assistants (n=210)	Dental clinics and hospitals	21-Item Depression Anxiety Stress Scale	DAs who were in direct contact with COVID-19 patients had higher distress symptoms. 41.43% of them felt downhearted during the pandemic. Mean total DASS was higher in men compared to women (p<0.05). depression and stress scores were higher in Saudi DAs compared to non-Saudi (p<0.05).	Dental assistants who have frequent patient contact are more likely to become infected, which heightens their psychological stress and worry.<br><br>3 – Moderate Low

Supplementary table
Table 2 Key Determinants of Dental Nurses' Health and Well-being of Included Studies
Colour code key - Pre Covid-papers - blue heading
Para Covid papers – Grey heading/Borders
Para pandemic papers that did not focus on the pandemic- Red borders
Papers where dental nurses cannot be separated – Yellow highlight

		Micro level factors			Meso level factors		Macro level factors		
**Study Author/ Date**	**Professional group**	**Personal Factors**	**Professional/ Social relationships**	**Professional Career Level**	**Job Specification**	**Workplace Characteristic**	**Dental Healthcare System**	**Regulation**	**Profession**
NAIDU, R. S. et al., 2002	Dental nurse	Career breaks due to family reasons	Childcare responsibility	Unsatisfied by career development		Job satisfaction linked to unsatisfactory work conditions	Confused about legislation		Dissatisfied with CEs available to them
ALANKO, K et al., 2004	Dental nurses					Occupational hazard- dermatoses			
LINDFORS, P. et al., 2006	Dentists, dental hygienists, and dental nurses	General health problems, increase age linked to UED				UED linked to poor physical, psychosocial work environment			
NAIDU, R. et al., 2006	Therapists-UK, NZ dental nurses-T&T					Lowest job satisfaction in nurses-Trinidad and Tobago			
JAAKKOLA, M. et al., 2007	Dental assistants	Atopic disease				Exposure to methacrylate at work			
HILT, B. et al., 2009	Dental assistants					Cognitive effects from occupational exposure to mercury			

		Micro level factors			Meso level factors		Macro level factors		
**Study Author/ Date**	**Professional group**	**Personal Factors**	**Professional/ Social relationships**	**Professional Career Level**	**Job Specification**	**Workplace Characteristics**	**Dental Healthcare System**	**Regulation**	**Profession**
KHADER, Y. S. et al., 2009	Dental assistants					Fairly satisfied with their job			
WIJARANAPHITI, S. et al., 2009	Dental nurses		Need to be accepted by society	Desire to reach Self-actualization	Stress caused by role conflict	Motivation liked to better performance			
DAJPRATHAM, P. et al., 2010	Clinical instructors, postgraduate students and dental assistants					Occupational hazard- musculoskeletal problems like pain, which affected health			
AZODO, C et al., 2011	Dentists, dental nurses, dental technologists, dental therapists, record officers					Workplace violence linked to long waits, cancellations, intoxication, and bills			
SAMAT, R. A. et al., 2011	Dentists, dental nurses, dental technicians, and dental surgery assistants					Compromised posture was linked to back pain			
MINAMOTO, K. et al., 2012	Dentists, hygienists, technicians, assistants, and receptionists	Young age linked to eczema				Occupational Hazard – Eczema			

		Micro level factors			Meso level factors		Macro level factors		
**Study Author/ Date**	**Professional group**	**Personal Factors**	**Professional/ Social relationships**	**Professional Career Level**	**Job Specification**	**Workplace Characteristics**	**Dental Healthcare System**	**Regulation**	**Profession**
TURNER, S. et al., 2012	Dental nurses			CPD increased clinical responsibilities and skills	Majority felt mandatory CPDs had not influenced role	Feeling respected, more involved, and important due to extended duties	Negative financial impact of mandatory registration on career	Registration fee too high Agree with compulsory registration and CPD	Issues with the funding of continuing professional development (CPD)
Yousra H.AL JAZAIRY et al., 2014	Dental assistants	Females satisfied compared to men	satisfied with their professional and personal life	degree linked to satisfaction	Satisfied assisting dentists	Those in private practice and those working less were more satisfied	Low income		
GNICH, W. et al., 2014	Extended duty dental nurses				Extended duty dental nurses (EDDNs) were satisfied with their role	Barriers and facilitators of duties			
HAKANEN, J. et al., 2014	Dentist/nurse dyads		Friendly dentists-to exhaustion in nurses						
GOETZ, K et al., 2016	Dental nurses		Satisfied with colleagues	long length of employment	Clear responsibility within team	Satisfied by job variety, working less linked to improved well being			
AL-OMOUSH, S et al., 2019	Dentists, dental technicians, and dental assistants	Hearing affected by age				Dental Assistants had poorer hearing in left ear			

		Micro level factors			Meso level factors		Macro level factors		
**Study Author/ Date**	**Professional group**	**Personal Factors**	**Professional/ Social relationships**	**Professional Career Level**	**Job Specification**	**Workplace Characteristics**	**Dental Healthcare System**	**Regulation**	**Profession**
HAMASHA, A. et al., 2019	Dentist, dental assistant, hygienists, dental lab tech	Non-Saudis were more satisfied than Saudis				Quality of service linked to satisfaction	Low satisfaction with income		
SCEPANOVIC, D. et al., 2019	General dentists, dental specialists, dental technicians, and dental assistants	Musculoskeletal pain was more common in women than in men							
UZIEL, N. et al., 2019	Dental assistants					Stress was linked to income, workload, and work hazards			
ALZAHEM, A et al., 2020	Dentists, dental assistants, Lab technicians and dental hygienists	Burnout linked to age, sex, and marital status.		Low accomplishments					
MAHENDRAN, K. et al., 2020	Administrative, dental nurses, dental technicians, and dentists	Fears of the virus on personal health, high anxiety	Self-isolation from family and social distancing due to Covid -19	Uncertainty of impact of Covid -19 on training and career progression	Changing work patterns, outside scope, and higher risk settings due to Covid -19	PPE scarcity			
PARTIDO, B. et al., 2020	Dentists and dental assistants					Poor ergonomics was linked to muscular pain			

PARA COVID PAPERS									
		**Micro level factors**			**Meso level factors**		**Macro level factors**		
**Study Author/ Date**	**Professional group**	**Personal Factors**	**Professional/ Social relationships**	**Professional Career Level**	**Job Specification**	**Workplace Characteristics**	**Dental Healthcare System**	**Regulation**	**Profession**
RADFORD, P. & MCKAY, A., 2020	Dental nurses			Training and CPD	Recognise their vital role	'Referrals made by dentists', 'parental pressure' and 'demands on nurses			
DREHER, A. et al., 2021	Dental assistants	Old age was linked to increase in perceived risk of infection	Family with the virus was linked to feeling PPE was inadequate		Lack of information and preparation during Covid-19	Increased risk of COVID-19, lack of PPE, Financial uncertainty during Covid-19			
FREEMAN, R. et al., 2021	Dental trainees and primary dental care staff	Depressive symptoms were less emotional exhausted				High quality service linked to emotional fatigue			
HUMPHRIS, G. et al., 2021	Vocational dentist, therapist hygienist, Trainee Nurses, dental nurses	Emotional exhaustion				Some primary care staff were not prepared to take care of their health and stress	Financial concerns for practice owners due to Covid-19	Lack of control of redeploying	Anxiety and sadness from low professional recognition
KIRLI, M. & KIRLI U., 2021	Dentists, nurses, data entry/cleaning staff					Increased anxiety restarting procedures			
MEKHEMAR, M. et al., 2021	Dental nurses	Psychological impact of pandemic	Lower anxiety in single people			Private clinic higher psychological impact			

		Micro level factors			Meso level factors		Macro level factors		
**Study Author/ Date**	**Professional group**	**Personal Factors**	**Professional/ Social relationships**	**Professional Career Level**	**Job Specification**	**Workplace Characteristics**	**Dental Healthcare System**	**Regulation**	**Profession**
PLESSAS, A. et al., 2021	Dentists and dental nurses		UDCs strengthened working relationship			Using strategies to maximize efficiency during Covid-19 challenges	Undervalued and ignored during COVID-19	Worries about litigation in covid-19 role	Dissatisfied with communication from chief dental officer
SANDHU, B. K. et al., 2021	Dental nurses, dentists, dental core trainees		Isolation from family, but good teamwork during COVID-19		New skills, knowledge gained via redeployment during covid-19	Constantly changing guidance, lack of suitable personal protective equipment			
UHLEN, M. et al., 2021	Dental specialists, general dental practitioners, dental hygienists, and dental assistants	Female participants concerned about Instability and Infection	Socially distanced from family and friends due to job responsibility			Increased work experience was associated with not expressing fear about instability and being infected and infecting others			
WANG, Y. et al., 2021	Dental nurses	Age and exercise linked to work engagement		Higher professional titles liked to higher work engagement					
ZHANG, Y et al., 2022	Dentists and dental nurses	Older age was linked to psychologic distress				Low income, burn out, linked to psychological distress			

BANAKAR, M. et al., 2023	Dentists, dental hygienists, dental assistants, and dental students	COVID-19 anxiety				Consecutive shifts and assistants linked to high PTSD symptoms			
KOTHA, S. B. et al., 2023	Dental assistants	Depressive symptoms				Work was challenging during the Covid-19 pandemic			
